# Anti-Inflammatory Effects of Cannabinoids in Therapy of Neurodegenerative Disorders and Inflammatory Diseases of the CNS

**DOI:** 10.3390/ijms26146570

**Published:** 2025-07-08

**Authors:** Dorota Tomaszewska-Zaremba, Alina Gajewska, Tomasz Misztal

**Affiliations:** Department of Animal Physiology, The Kielanowski Institute of Animal Physiology and Nutrition, Polish Academy of Sciences, 05-110 Jablonna, Poland; a.gajewska@ifzz.pl (A.G.); t.misztal@ifzz.pl (T.M.)

**Keywords:** cannabinoids, endocannabinoids, neurodegenerative disorders, inflammatory diseases of the Central Neurvous System (CNS)

## Abstract

Many neurodegenerative diseases are associated with immune system disorders, while neurodegenerative processes often occur in inflammatory conditions of the Central Nervous System (CNS). Cannabinoids exhibit significant therapeutic potential due to their dual ability to modulate both neural and immune functions. These compounds have a broad spectrum of action, allowing them to target multiple pathological mechanisms underlying neurodegenerative and inflammatory CNS diseases. The present review outlines the therapeutic potential of cannabinoids, with a focus on their anti-inflammatory properties, in the treatment of neurodegenerative conditions, including Alzheimer’s disease, Parkinson’s disease, amyotrophic lateral sclerosis, and Huntington’s disease, as well as inflammatory CNS disorders like multiple sclerosis and HIV-associated dementia.

## 1. Background

Recent research has established a strong connection between neurodegenerative diseases and inflammatory processes, demonstrating that inflammation plays a significant role even in non-inflammatory Central Nervous System (CNS) disorders. Conversely, classical inflammatory CNS conditions frequently display neurodegenerative characteristics. Key neurodegenerative diseases that are strongly associated with inflammation include Alzheimer’s disease (AD), Parkinson’s disease (PD), amyotrophic lateral sclerosis (ALS), and Huntington’s disease (HD), while inflammatory CNS disorders leading to neurodegenerative processes encompass multiple sclerosis (MS) or HIV-associated dementia [1]. This interplay between neuroinflammation and neurodegeneration has created a demand for therapeutic approaches that combine neuroprotective and anti-inflammatory effects. Cannabinoids (CBs), whether natural, synthetic, or endogenous (endocannabinoids, ECBs), are compounds that are capable of simultaneously modulating immune responses and neurodegenerative processes [2]. This review explores the potential application of cannabinoids, with a particular focus on their anti-inflammatory properties in the treatment of the illnesses discussed.

## 2. Neurodegenerative Disorders and Inflammation

**Figure 1:**
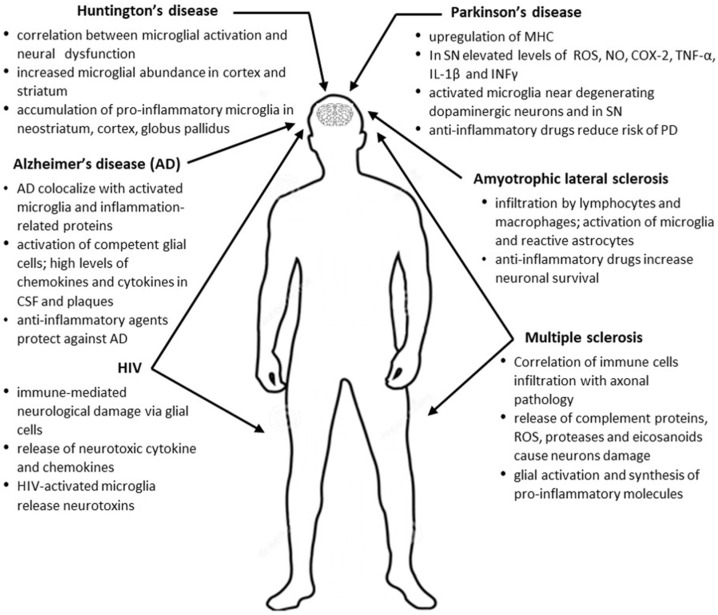
Neurodegenerative disorders and inflammatory diseases of the CNS and inflammatory processes.

There is substantial evidence that inflammation is inherent in many primarily non-inflammatory CNS diseases, including neurodegenerative disorders. Although inflammation is not regarded as the root cause of these conditions, growing evidence suggests that CNS damage during neurodegeneration is influenced by immunological mechanisms (Figure 1). The controversy stems from the fact that inflammation may also exert neuroprotective effects. In neurodegenerative disorders, both clinical evidence, including therapies targeting inflammatory processes, and animal studies indicate a significant impact of inflammation on disease pathology. A key role in neuroinflammation is attributed to glial cells, specifically astroglia and microglia. Gliosis is a hallmark of neurodegenerative diseases, and neuroinflammation, primarily mediated by glial cells, is a characteristic feature of many neurodegenerative and demyelinating disorders, such as AD, PD, ALS, and MS [3,4,5]. Glial cells, now recognized for their significant role in brain physiology, metabolism, development, and nervous system diseases, are essential for maintaining the stability and integrity of the nervous system [6,7]. Several types of glial cells present in the CNS can be distinguished: astrocytes, oligodendrocytes, and microglia. Among these, astrocytes are the most abundant, comprising approximately 50% of cells in the CNS and spinal cord in humans. The distinctive star-shaped morphology of astrocytes, with numerous long and branching processes, allows them to closely interact with neurons, enabling efficient metabolic coordination and support [8]. Astrocytes are central regulators of neuroinflammatory processes in the CNS, and are activated in response to injury and disease. Depending on the timing and context, their activity may either exacerbate inflammatory reactions and tissue damage or promote immunosuppression and tissue repair. Transforming growth factor beta (TGFβ), interferon gamma (IFNγ), glycoprotein (gp130), estrogen, signal transducer and activator of transcription 3 (STAT3), brain-derived neurotrophic factor (BDNF), and Fas ligand (FASL) contribute to the protective phenotype of astrocytes, whereas interleukin 17 (IL-17), sphingolipids, tyrosine receptor kinase B (TrkB), suppressor of cytokine signaling 3 (SOCS3), nuclear factor kappa B (NF-κB), chemokines, and vascular endothelial growth factor (VEGF) activate pathways associated with cellular damage [5,9]. Oligodendrocytes are distributed throughout the gray and white matter in the CNS. The cell body of oligodendrocytes is round or oval, with fewer protrusions wrapped around neuronal axons. Myelinating oligodendrocytes originate from oligodendrocyte progenitor cells (OPCs), which are still present in the adult CNS. The capacity of OPCs to proliferate and differentiate into myelinating oligodendrocytes is critical in the development of neurological autoimmune disorders, e.g., MS [10]. Microglia are the resident immune cells accounting for 5–10% of the total CNS cell population in humans. These cells possess a small, short, rod-shaped body with long, thin protrusions. Depending on their function and status, microglia can be defined as process-bearing, highly ramified myeloid cells and tissue-resident macrophages [11]. In a healthy brain, microglia are highly mobile due to their dynamic processes, actively monitoring the microenvironment for signs of damage and debris [12]. As functionally diverse cells, microglia are regulated by various signaling pathways, including Toll-like receptors [13], NFkB [14], mitogen-activated protein kinases (MAPKs) [15], Janus kinases/signal transducer and activator of transcription proteins (JAK/STAT) [16], CX3C motif chemokine receptor 1 (CX3-CX3CR1) [17], and peroxisome proliferator-activated receptors (PPARs) [18]. Microglial activation, leading to the induction of pro-inflammatory factors such as IL-1β and tumor necrosis factor α (TNF-α), has been shown to trigger neurodegeneration. IL-1 β and TNF-α are involved in the development of CNS inflammation through the induction of adhesion molecules and chemokines. Several microglial ion channels, including potassium, calcium, chloride, sodium, and proton channels, have been proposed as potential drug targets in many neurodegenerative disorders. These channels play a role in regulating microglial functions such as proliferation, chemotaxis, phagocytosis, antigen recognition and presentation, apoptosis, and cell signaling, all of which contribute to inflammation [19]. On the other hand, microglia are also source of anti-inflammatory agents, which are able to counterbalance inflammation and promote their homeostatic state. By releasing anti-inflammatory cytokines and growth factors, microglia exhibit neuroprotective functions, thus preventing mitochondrial damage and neurodegeneration [20,21]. These contradictory roles of microglia might be explained by the presence of distinct microglial subpopulations having different molecular signatures and functions [22]. In their resting state, inactivated microglia secrete neurotrophins and clear debris from dead cells, and detect soluble factors released by neurons, astrocytes, other microglia, and infiltrating peripheral immune cells [23]. Additionally, microglia can transit into either an activated M1 (pro-inflammatory) or M2 (anti-inflammatory) phenotype [24]. M1 microglia exhibit regulated phagocytic activity and promote the release of pro-inflammatory cytokines which, at sufficient concentrations, trigger neuronal signaling cascades leading to cell impairment or necrosis [25]. In a healthy brain, the inflammation process is stopped before cell damage occurs, and M1 microglia transit toward a more anti-inflammatory M2 phenotype to release cytokines [26,27]. Failure to control the inflammatory response and engage M2 microglia is a common mechanism underlying neurodegeneration [27]. More recently, however, activated microglia have been shown to exist across a diverse spectrum of functional states, and it is likely that their response to neuroinflammation results from the simultaneous presence and activity of multiple microglial phenotypes [28,29]. Evidence of induction of a specific transcriptional network that specifies microglial identity in a pathology-dependent environment has been recently shown in the human CNS [30].

The blood–brain barrier (BBB) is a specialized protective vascular structure that separates the blood from the brain’s extracellular fluid. It regulates cerebral blood flow and ensures the transport of oxygen, glucose, and essential metabolites necessary to support CNS nourishment and maintain homeostasis. The endothelial cells that form the BBB blood vessels, capillaries, and their basement membranes possess specific structural and functional characteristics (such as the absence of fenestration and the presence of tight junctions, as well as the use of active transport mechanisms) that are essential for tight control of compound transport and cellular trafficking into and out of the CNS [31]. The BBB endothelium operates within the “neurovascular unit” composed of neurons, mural cells (pericytes and smooth muscle cells), astrocytes, and microglia [32]. Highly restrictive and selective molecular permeation through the BBB paracellular route depends predominantly on the presence of endothelial junctions, including adherens junctions (AJs) and tight junctions (TJs). TJs consist of integral membrane proteins, such as occludins and claudins, operating together with the cytoplasmic accessory proteins zonula occlude ZO-1 and ZO-2 [33,34]. There are data indicating that cannabinoids might be involved in the regulation of BBB permeability through junction protein complexes [35]. For example, studies in vitro on human microvascular endothelial cells have revealed that cannabinoid agonists could prevent the downregulation of TJ proteins [36]. In mice, cannabinoids operating via CB2 receptors have been shown to suppress inflammation, prevent BBB damage, and attenuate an increase in the expression level of intercellular adhesion molecule-1 (ICAM-1) [37]. Recently, a functional connection between astrocytic endocannabinoid system activity and the BBB has been observed in male mice. In that study, high expression of endocannabinoid CB1 receptor in the nucleus accumbens (nAc) shell was reported to both promote vascular-related gene expression and reduce the astrocyte inflammatory response in association with resilience during chronic stress [38].

Under physiological conditions, microglia regulate BBB properties by maintaining a delicate balance between proper neuronal activity and interactions with other components of the neurovascular unit. The influence of microglial cells on BBB functions begins early in embryogenesis, where they have been shown to contribute to cortical neurogenesis, as well as vascular [39] and visual system development [40]. In the adult CNS, microglia make transient and dynamic contact with the neurovasculature, enabling them to monitor vascular integrity and respond to changes in the microenvironment [41].

### 2.1. Alzheimer’s Disease

Alzheimer’s disease, the most common cause of dementia, is a neurodegenerative disorder characterized by distinct pathological features, such as amyloid-β (Aβ) plaques, tau protein accumulation, neurofibrillary tangles (NFTs), and oxidative damage in neurons, microglia, and endothelial cells. Activated microglial cells release pro-inflammatory cytokines such as IL-1, TNF-α, and IL-6, which contribute to neuronal degeneration [42,43]. In AD patients, Aβ has been shown to colocalize with activated microglia and inflammation-related proteins [44]. Further evidence of neuroinflammation includes the activation of complement and glial cells, along with elevated levels of acute-phase proteins, chemokines, and cytokines in cerebrospinal fluid (CSF) and plaques [45]. Genetic studies also implicate polymorphisms in pro-inflammatory cytokine or acute-phase protein genes as risk factors for AD [46]. Recently, in an animal model for Alzheimer’s disease, microglial clusters with distinct gene expression profiles in cortical regions were identified, indicating more diverse microglia states during disease progression. These “disease activated microglia” (DAM) were present in two distinct stages: the transition from the normal state to stage 1 was TREM2-independent, whereas the further shift from stage 1 to 2 required TREM2 signals [47]. Growing evidence suggests that anti-inflammatory agents may exert protective effects against AD. Notably, epidemiological studies have demonstrated that patients receiving high doses of non-steroidal anti-inflammatory drugs (NSAIDs) for conditions such as rheumatoid arthritis or cardiovascular disease show a reduced risk of developing AD [48].

### 2.2. Parkinson’s Disease

Parkinson’s disease is a complex, age-related neurodegenerative disease associated with dopamine deficiency and a combination of motor and non-motor symptoms. The neuropathological hallmarks of PD include the presence of Lewy bodies and Lewy neurites, leading to neuronal degeneration in the substantia nigra (SN) and other vulnerable brain regions. The pathology of PD includes depigmentation and selective neuronal loss in the SN and locus coeruleus, driven by mechanisms such as apoptosis, autophagy, mitochondrial dysfunction, and oxidative stress. The first report linking inflammatory processes to PD was published in 1988, showing upregulation of major histocompatibility complex (MHC) molecules in the brains of PD patients [49]. Later research demonstrated elevated levels of reactive oxygen species (ROS), nitric oxide (NO), cyclooxygenase (COX)-2, tumor necrosis factor (TNF)-α, IL-1β, and IFN-γ in the SN in these patients [49]. Activated microglia have been observed near degenerating dopaminergic neurons in PD cases, and microglial activation in the SN has been shown to cause neuronal damage [50]. In addition, studies have indicated that certain cytokine polymorphisms may increase the risk of PD [51]. Experimental models of PD utilizing dopamine neuron-specific neurotoxins show microglial activation in nigral and striatal microglia, along with increased production of pro-inflammatory molecules. Microglial inhibition has been demonstrated to exert a neuroprotective effect in experimental models, further supporting the role of neuroinflammation in PD [52]. Interestingly, rodent studies have demonstrated that a pro-inflammatory stimulus can also induce neuroprotection, with concomitant reductions in microglial activation and increased cytokine levels at sites of neurodegeneration [53]. Epidemiological studies, on the other hand, have indicated that chronic treatment with anti-inflammatory drugs, such as NSAIDs, reduces the risk of PD by approximately 45% compared to non-regular NSAID users [46].

### 2.3. Amyotrophic Lateral Sclerosis

ALS is a progressive neurodegenerative disorder characterized by the degeneration of motor neurons, resulting in impairments of essential functions such as swallowing, speaking, and breathing [54]. Strong inflammatory responses have been observed in the brains of ALS patients, with evidence showing that these processes intensify with disease progression [55]. Experimental studies using rat spinal cord neurons treated with ALS cerebrospinal fluid demonstrated that pharmacological suppression of microglial activation increased neuronal survival [56]. Research on transgenic mice expressing human superoxide dismutase 1 (SOD1) with a G93A mutation (hSOD1G93A), a well-established animal ALS model, showed that the administration of anti-inflammatory drugs prolonged the survival of these mice [57]. Clinical trials have also been conducted on the effect of immunomodulatory drugs in people with ALS [58].

### 2.4. Huntington’s Disease

Huntington’s disease is a neurodegenerative disorder characterized by distinctive choreatic movements, motor dysfunction, dementia, and cognitive decline. The degenerative process primarily affects medium spiny striatal neurons and cortical neurons, leading to dysfunction and subsequent neuronal loss [59]. The pathophysiology of HD, while not fully understood, is known to be an autosomal dominant neurodegenerative disorder caused by a CAG trinucleotide repeat expansion in the HTT gene on chromosome 4. This mutation results in an abnormal polyglutamine stretch in the huntingtin protein, leading to progressive neurodegeneration, followed by motor and cognitive impairments [60]. Growing evidence indicates neuroinflammation in HD pathogenesis, with activated microglia playing a central role. Studies demonstrate a direct correlation between microglial activation and neuronal dysfunction, supported by observations of increased microglial abundance in the cortex and striatum of HD patients. This inflammatory response progresses with disease stage, showing accumulation of pro-inflammatory microglia in the neostriatum, cortex, and globus pallidus [61], and a marked increase in the binding of the PK11195 ligand. This compound binds selectively to peripheral benzodiazepine binding sites, receptors selectively expressed by activated microglia, in the cortical and striatal regions of HD patients [62,63]. Activation of the immune system and an altered immune response have been observed even in the premanifest stage of the disease [64]. However, inflammation in HD may be the consequence of neuronal death induced by the mutated huntingtin protein [59]. Mitochondrial dysfunction, a known pathological factor in HD, may also contribute to inflammatory processes [65].

Cannabinoids, as well as agents that modulate cannabinoid receptor activity, may offer a unique opportunity to simultaneously address inflammation and neurodegeneration. Cannabinoid receptors are widely expressed in neurons and in immune cells of the central and peripheral nervous systems, where they play key roles in regulating neurodegenerative and inflammatory processes.

## 3. Inflammatory Diseases of the CNS and Neurodegeneration

Inflammatory processes in the CNS trigger a cascade of immune-mediated damage that can lead to neurodegeneration. Although neurons lack MHC expression, they frequently sustain collateral damage during inflammatory attacks targeting glial cells. Paradoxically, certain autoimmune inflammatory responses may also exert neuroprotective effects against degenerative processes [66].

### 3.1. Multiple Sclerosis

MS is a chronic disease involving demyelination of CNS neurons, resulting from complex genetic and environmental interactions. Current understanding identifies MS as an immune-mediated disorder involving both the cellular and humoral immune systems [67]. Neuropathological examinations have revealed early axonal pathology in MS patients, strongly correlated with immune cell infiltration [68]. Neurodegenerative processes have already been observed during early MS stages, partly because of axonal demyelination. Various mechanisms are responsible for these processes, including axonal transection, cytotoxic T cell activity, and damage caused by soluble products released by resident and invading inflammatory cells, such as axon-specific antibodies, complement proteins, NO, oxygen radicals, proteases, or eicosanoids [69]. The characteristic demyelination observed in MS results primarily from T cell-mediated autoimmune responses. The main pathological factors include leukocyte infiltration into the CNS, followed by glial activation and the production of inflammatory molecules such as TNF-α, IFN-γ, NOS, COX-2, glutamate free radicals, and NFkB. scRNA-seq analysis performed in microglia isolated from patients with MS has revealed three common microglia clusters that express homeostatic microglia genes such as TMEM119, P2RY12, and SLC2A5, and one common cluster with high expression of the chemokines CCL2 and CCL3 [70]. Interestingly, T cells may also exert neuroprotective effects by producing neurotrophins [46].

### 3.2. HIV-Associated Disease

The precise mechanisms underlying immune-mediated neuronal damage via glial cell alterations in HIV-associated dementia remain unclear. Current evidence indicates that the release of neurotoxic cytokines and chemokines plays an important role in this process. Additionally, HIV-activated microglia release neurotoxins such as arachidonic acid, glutamate, TNF-α, and IL-1, which can induce neuronal damage [1].

## 4. Cannabinoids and the Immune System

The endocannabinoid system plays a key role in maintaining homeostasis in an organism and regulates various physiological processes, including inflammation. ECBs are lipid-based mediators, isolated from brain and peripheral tissues, that comprise amides, esters, and ethers of long-chain polyunsaturated fatty acids [71]. The best-characterized ECBs are anandamide (AEA) and 2-arachidonylglycerol (2-AG). Unlike classical neurotransmitters, ECBs are not stored in vesicles or cells, but are synthesized on demand from membrane lipid precursors following calcium-dependent enzymatic activation [72]. These lipid mediators exert their effects primarily through cannabinoid receptors (CBRs), including CB1 receptor (cloned by Matsuda in 1990) and CB2 receptor (identified by Munro). They are members of the G protein-coupled receptor superfamily, which is characterized by seven transmembrane domains [73]. CB1 receptors are predominantly expressed in the CNS, where they mediate most of the central effects of CBs. Peripherally, CB1 receptor expression has been found in the pituitary gland, immune cells, reproductive and gastrointestinal tissues, superior cervical ganglion, blood vessels, lung, bladder, adrenal gland, liver, and adipose tissue [74]. In contrast, CB2 receptors were initially thought to be exclusively peripheral, with predominant expression in immune cells (B cells and natural killer cells), the spleen, the thymus, the tonsils, splenic macrophage/monocyte preparations, mast cells, and circulating leukocytes. However, recent studies have identified CB2 receptor expression in CNS cell populations, including microglia, brain stem cells, and specific brain regions such as the cerebellum, striatum, midbrain, and hippocampus [74]. Additionally, pharmacological evidence indicates the existence of other CB receptors that differ from CB1 and CB2 receptors. These include G protein-coupled receptors such as GPR55 and GPR119, as well as the transient receptor potential cation channel subfamily V member 1 (TRPV1) [74]. Both types of cannabinoid receptors are present on immune cells, with their expression levels modulated by the organism’s physiological state, such as infection or immune activation. Pro-inflammatory cytokines (TNF-α, IL-1β, and IL-6) have been shown to upregulate both receptor types in human peripheral blood mononuclear cells and T lymphocytes [75]. CB2 typically demonstrates higher expression in the immune system than CB1, being particularly abundant in B cells, NK cells, monocytes, neutrophils, and T cells [76]. Dendritic cells also express CB2, suggesting a modulatory role of cannabinoids in antigen presentation [77]. In the CNS, microglia express both receptor subtypes, with CB2 showing marked upregulation during microglial activation [78].

Exogenous, endogenous, and synthetic cannabinoids are widely recognized as regulators of the immune system. Their effects on immune cells can be either stimulatory or suppressive, depending on the receptor type, concentration, and target cell population. In vitro studies have demonstrated a concentration-dependent biphasic response: nanomolar concentrations typically stimulate immune function, while micromolar concentrations generally inhibit it [79]. Generally, cannabinoids inhibit cell proliferation, reduce cytokine and chemokine secretion, and suppress bone marrow-derived myeloid cell recruitment, while promoting regulatory T cell differentiation and apoptosis [80]. The relationship between cannabinoids and cytokines is very interesting due to its bidirectional nature. Cannabinoids can modulate cytokine secretion, which in turn affects cannabinoid receptor activity. This interplay can shift cytokine profiles from pro-inflammatory to anti-inflammatory states [80]. For example, both synthetic and plant-derived cannabinoids inhibit TNF-α and other acute-phase cytokines, although in certain conditions, they can increase the expression of TNF-α and other pro-inflammatory cytokines and chemokines [81]. In immune cells, cannabinoid receptor activation regulates DNA-binding of different nuclear factors, leading to reduced cAMP production through adenylate cyclase inhibition. On the other hand, short-term, rapid bursts of adenylate cyclase activity are associated with preceding lymphocyte activation by mitogens, while cytokine transcription in macrophages is regulated via the cAMP signaling cascade [75].

CBR stimulation antagonizes the regulatory role of the cAMP pathway during early immune cell activation [76]. In addition to cAMP-mediated effects, cannabinoid receptors can act through Gi proteins and exert a dual effect on MAPK activity, depending on the specific ligand and cell type [75]. Data from the literature indicate that MAPK pathways play a role in the interaction between cannabinoid signaling and inflammatory processes. MAPKs are intracellular signaling proteins that are subdivided into c-Jun N-terminal kinase (JNK), extracellular signal-regulated kinase (ERK), and p38 proteins. Interestingly, each of these pathways has been associated with both the pro- and anti-inflammatory properties of microglia [82]. Collectively, these findings indicate that the endocannabinoid system is a key regulator of immune system activity, implying that therapeutic modulation of this system will inevitably produce immunological consequences [83].

Endocannabinoids like 2-AG and AEA can serve as sources of arachidonic acid (AA), which is metabolized by eicosanoid biosynthetic enzymes to generate various bioactive lipids. These AA-derived metabolites exhibit either pro-inflammatory or anti-inflammatory properties. Importantly, pharmacological inhibition of endocannabinoid degradation may elevate the levels of these immunomodulatory lipids, thereby influencing inflammatory cell activity [84].

**Figure 2:**
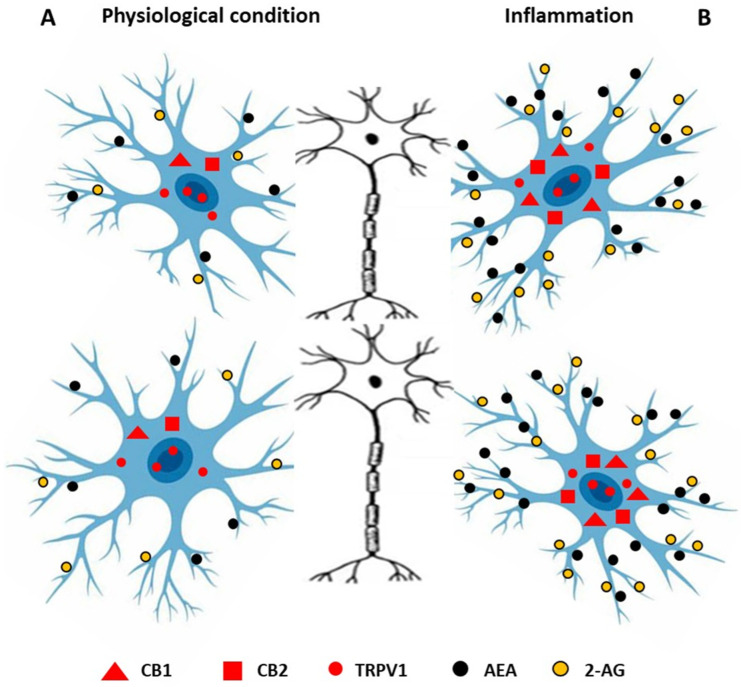
Endocannabinoids and their receptors in microglia under physiological conditions (**A**) and during inflammation (**B**). Under physiological conditions, microglia express TRPV1 and low levels of CB1 and CB2, and secrete a small amount of anandamide AEA and 2-arachidonylglycerol (2-AG). During inflammation, microglia release high levels of AEA and 2-AG, and the expression of CB1 and CB2 increases.

Cannabinoids interact with the immune system not only in peripheral tissues, but also within the brain, playing an important role in maintaining the balance between neuroinflammatory processes and neurodegenerative pathways. Microglia possess the complete molecular machinery for endocannabinoid synthesis, metabolism, and signaling [85,86,87]. Under physiological conditions, microglia produce 2AG and AEA, while expressing cannabinoid receptors at low levels. CB1 receptors are widely distributed throughout the CNS, primarily on neurons, and are expressed at constitutively low levels in microglia cultured from mice and rats; however, their presence has not been detected in human microglia. CB2 receptors are also coupled with Gi/o proteins and are expressed in microglia cultured from mouse, rat, and human tissues [85,88]. Inflammatory activation of microglia increases their synthesis of ECBs and upregulates the expression of CB2. This CB2-mediated signaling promotes a neuroprotective microglial phenotype characterized by increased secretion of trophic factors and decreased production of pro-inflammatory mediators [89]. Activation of CB2 receptors in microglia leads to increased microgliosis, migration, and phagocytosis, while suppressing the synthesis of pro-inflammatory mediators such as TNF-α and free radicals [89]. Through these mechanisms, cannabinoids modulate microglial activation states, favoring a transition from pro-inflammatory to anti-inflammatory phenotypes via multiple signaling pathways (Figure 2). The regulation of CBRs and other components of the endocannabinoid system in microglia under inflammatory conditions depends on the type of stimuli and the duration of exposure. ECBs exert anti-inflammatory effects and facilitate communication between glia and neurons through CB1 and CB2 receptor activation [90]. During neuroinflammation, CB2 receptor mRNA expression can increase dramatically—up to 100-fold in some cases. For example, in mice with experimental autoimmune encephalitis and in IFN-γ/LPS-stimulated immortalized N9 microglia, CB2 mRNA levels rose 12-fold [91]. However, in primary rat microglia exposed to LPS, the components of the endocannabinoid system were downregulated [92]. Stimulation of CB1 receptors by synthetic cannabinoids has been shown to suppress the synthesis of pro-inflammatory cytokines in glia, demonstrating anti-inflammatory effects in vitro [93,94]. Notably, microglial regulation of pro-inflammatory cytokine secretion may also involve pathways independent of CB1 and CB2 receptors. GPR55, identified in microglia, can be activated or inhibited by various cannabinoids [89]. Astrocytes, another type of glial cell, play a key role in neuroinflammation by modulating blood–brain barrier permeability and controlling immune infiltration into the brain. Astrocytes also function as immune cells by releasing cytokines and chemokines and activating adaptive immune responses during inflammation [95]. These cells contain elements of the endocannabinoid system, including AEA, 2-AG, their receptors and related enzymes [95]. Most research on how cannabinoids affect astrocyte-related inflammation has been conducted using astrocyte cultures. However, in our earlier study on ewes, we also demonstrated the anti-inflammatory properties of AEA, which interfered with interleukin-1β (IL-1β) synthesis and IL-1 system gene expression in hypothalamic structures during immune challenges. AEA inhibited LPS-stimulated synthesis of central IL-1β in the hypothalamus, likely affecting posttranscriptional levels of this cytokine. The anti-inflammatory effect of AEA in the CNS may also involve increased expression of the IL-1RN and IL-1R2 genes [96].

Aging has been demonstrated to correlate with alterations in ECB system tone, with CB1 expression peaking in adolescence and decreasing with age [97]. Studies by Dvir-Ginzberg et al. [90] in mice showed that the loss of CB1 receptors in the hippocampus accelerated local neuroinflammatory progression in adult mice. Furthermore, conditional CB1 receptor knockout in the ventromedial hypothalamus (VMH) specifically was shown to potentiate pro-inflammatory responses. This was evidenced by greater microglial and astrocyte density, along with elevated TNFα signaling. Conversely, restoring CB1 receptor expression mitigated these age-related inflammatory changes.

The endocannabinoid system represents a promising therapeutic target for neuroinflammation, supported by substantial evidence demonstrating the ability of synthetic, natural, and endogenous cannabinoids to suppress the pro-inflammatory response of microglia and promote their shift toward an anti-inflammatory phenotype. Activation of CB2 has been proposed as the mechanism of action responsible for these effects [98]. The anti-inflammatory effects of cannabinoids are thought to be due specifically to their potential inhibitory effect on NLRP3. In addition, recent findings suggest that cannabinoids, being CB1 receptor agonists, play a key role in this anti-inflammatory mechanism of action. In addition, cannabinoids play a role in inflammatory cytokine signaling pathways [99]. This results in inhibition of GABA receptors, and thereby affects working memory; changes in the pain response also occur [100]. Experimental studies have also shown that CB2 receptors modulate glutamate release, but the precise mechanism is unclear [101]. Unlike those in CB1 receptors, changes in CB2 receptors in neuropsychiatric disorders have not been associated with major changes. An increase in the activity of these postsynaptic receptors inhibits neuronal activity and modulates CB1 receptors in the hippocampus.

## 5. The Anti-Inflammatory Potential of Cannabinoids in Neurodegenerative Disease Therapy

The key to effective therapy for neurodegenerative disorders lies in reducing inflammation by restoring the brain’s ability to regulate it. Controlling inflammation can lower the risk of developing neurodegenerative diseases [46]. This goal may be achieved through a combination of drugs targeting multiple factors contributing to inflammation, with cannabinoids and agents modulating cannabinoid receptor activity being promising candidates for such therapy [46]. Cannabinoids possess a broad spectrum of action, allowing them to affect many pathological changes characteristic of neurodegenerative diseases. Both exogenous and endogenous cannabinoids have been shown to exert neuroprotective effects in diverse in vitro and in vivo models of neuronal injury [1]. The use of cannabis in the treatment of neurodegenerative diseases is gaining increasing interest. In the late 20th century, the first cannabis-based drug was approved for clinical use and has since been used to treat neurological disorders. Clinical trials with CBD have begun to show promise for treating epilepsy, insomnia, and social anxiety [1]. Notably, nabiximols—a standardized extract containing tetrahydrocannabinol (THC) and cannabidiol (CBD)—has been approved for treating spasticity and neuropathic pain in MS, while purified natural cannabidiol is approved for managing various other neurological conditions. CBD exerts its antioxidant activity in two ways: directly, due to its chemical structure, and indirectly, through mechanisms controlling oxidative balance (redox homeostasis). CBD helps to reduce the production of ROS primarily through the property of chelating transition metal ions that enter the Fenton reaction and create free radicals. CBD increases the gene expression of the main endogenous antioxidant enzymes, such as superoxide dismutase (SOD) and glutathione peroxidase (GPx). This occurs through a process involving the nuclear erythroid 2-related factor (Nrf2)/Keap1 complex. Moreover, CBD helps to keep levels of zinc (Zn) and selenium (Se) stable—these elements are important for the proper function of SOD and GPx. One common effect of oxidative stress is lipid peroxidation, where fats are damaged and turned into harmful molecules like malondialdehyde (MDA) and 4-hydroxynonenal (4-HNE). These molecules can react with DNA, proteins, and fats, leading to further damage and lowering the protective glutathione/oxidized 12glutathione (GSH/GSSG) ratio (a marker of oxidative stress). Many studies show that CBD protects the brain from oxidative damage by lowering MDA levels. For example, in AD models, CBD has also been found to lower the amount of oxidized fats (PUFAs), showing its protective effect in the brain [102]. Additionally, cannabinoids have demonstrated efficacy in treating drug-resistant forms of childhood epilepsy, highlighting the great potential of this group of compounds for clinical applications in neurological diseases [103].

### 5.1. Alzheimer’s Disease

Cannabinoids, despite their controversial status, have emerged as promising candidates for AD therapy. Their action through CB1 and CB2 receptors targets multiple pathological processes involved in AD, including beta-amyloid deposition, tau protein phosphorylation, inflammation, mitochondrial dysfunction, and excitatory neurotoxicity. Stimulation of CB1, CB2, and other cannabinoid-reactive receptors has been shown to prevent microglial activation and microglia-mediated neurotoxicity and neurodegeneration in experimental AD models [104]. Similar neuroprotective effects can be achieved by elevating endogenous cannabinoid levels through inhibition of AEA cellular uptake [105]. Both cannabinoid receptor agonists and endocannabinoids such as AEA have been shown to mitigate Aβ-peptide-induced neurotoxicity through the MAPK pathway in a CB1-dependent manner, protecting human NTERA-2/cl-D1 teratocarcinoma cells [106]. Specific changes in the endocannabinoid system, such as CB2 receptor upregulation and dysregulation of 2-AG metabolism, have been reported in mouse AD models [60,107]. Enhanced CB2 receptor-like immunoreactivity has also been observed in plaque-associated microglia in humans [108,109]. In human AD brains, FAAH activity was found to be upregulated in plaque-associated glial cells [108], along with significant alterations in 2-AG metabolic enzymes. Isolated membrane and cytosolic fractions from this tissue showed an accelerated rate of 2-AG degradation compared to controls [107]. Moreover, a positive correlation between cognitive function and AEA levels in AD patients, in parallel with a negative correlation between AEA concentrations and Aβ42 abundance, suggests dysregulated AEA production in AD [110]. Overall, brains afflicted with AD exhibit impaired endocannabinoid signaling, likely resulting from enhanced degradation of endocannabinoids without a compensatory increase in synthetic enzymes.

Cannabinoids regulate the transition of microglia from a resting to an anti-inflammatory phenotype. In several AD models, CB2 receptor activation has been demonstrated to dampen neuroinflammation and improve cognitive performance [111,112]. Activation of microglial CB2 receptors has also been shown to stimulate phagocytosis of Aβ [113,114], indicating their dual role in promoting Aβ clearance and reducing neuroinflammation. These findings suggest that cannabinoids such as THC might be effective in AD therapy due to their diverse mechanisms of action. Recent studies involving animal AD models have demonstrated that THC supports hippocampal neurogenesis, prevents neurodegenerative processes, mitigates inflammation and cognitive impairment, and restores memory and cognitive functions [115,116,117]. Among natural cannabinoids, CBD has gained attention for its therapeutic potential in AD due to its favorable safety profile and limited systemic absorption when administered orally [115]. Experimental evidence indicates that this compound reduces reactive gliosis and neuroinflammatory responses [118], while stimulating neurogenesis [119].

### 5.2. Parkinson’s Disease

Aside from the degeneration of dopaminergic neurons in the SN and intraneuronal synuclein deposits, inflammation is a significant contributor to the pathology of PD. The inflammatory response in PD is predominantly mediated by microglial cells. Early research identified a close association between activated M1 microglia and neuronal damage in PD [120], with subsequent research confirming an elevated presence of M1 microglia in PD-affected brains [121,122]. The inflammatory process correlates strongly with disease progression, as evidenced by elevated populations of CD54/CD11a+ microglia producing TNFα and IL-6 in the SN and other vulnerable brain areas [123]. Microglial cytokine release contributes to dopaminergic neuron damage and death through neuroinflammatory mechanisms [124]. Conflicting data exist regarding the expression of CB1 and CB2 receptors in PD. In the human brain, elevated CB1 receptor mRNA levels were observed in the caudate putamen, whereas CB1 mRNA expression in the SN remained unchanged [125]. In non-human primates with levodopa-induced dyskinesia, increased CB1 receptor mRNA expression was detected in both the globus pallidus and subthalamic nucleus [126]. CB2 receptor expression shows distinct patterns, with increased mRNA in the SN but decreased levels in the caudate putamen in human [111] and mouse tissues [127]. Moreover, CB2 receptor protein expression was upregulated in activated microglia in the midbrain of PD model mice [128]. A substantial increase in the expression of the most prevalent endocannabinoid, 2-AG, was observed in the globus pallidus in reserpine-treated mice [129], and in the ventral midbrain of MPTP-treated mice [130]. The CB2 receptor agonist β-caryophyllene reduced the pro-inflammatory response of microglia in rats [131,132], while the synthetic agonist WIN55, 212-2 decreased neuronal death and improved motor symptoms in MPTP-induced neurotoxicity mouse models. Existing data suggest that enhanced endocannabinoid synthesis and activation of microglial receptor signaling exert neuroprotective effects against neuroinflammatory processes in PD [111]. Studies on experimentally induced PD have demonstrated that CB1, CB2, and non-CB1/non-CB2 receptor agonists reduce dopaminergic neuron degeneration by modulating the interaction between glial cells and neurons [133]. However, the activation of CB1 receptors has also been shown to exacerbate the toxic effects of the TRPV1 agonist capsaicin on dopamine cell survival [134]. This suggests that endocannabinoids like AEA, which activate both TRPV1 and CB1 receptors [135], may contribute to PD pathophysiology by promoting dopaminergic neuron apoptosis.

### 5.3. Amyotrophic Lateral Sclerosis

Research on cannabinoid therapies in ALS supports the relevance of CB2 receptor activation in modulating astrocyte trophic support, microglial reactivity, and neuroinflammation. Experimental studies using hSOD1G93A mice have revealed that both cannabinoid receptor agonists and elevated endocannabinoid levels through FAAH ablation [136] produce strong anti-inflammatory and neuroprotective effects, significantly slowing disease progression [137,138]. These beneficial effects appear to be predominantly mediated by CB2 receptor stimulation, while CB1 receptor activation has been associated with detrimental effects on motor neuron survival [138].

### 5.4. Huntington’s Disease

Numerous studies have reported immune system activation or altered immune response in HD, suggesting that treatments targeting the immune system could potentially alleviate symptoms of this disease. The correlation between HD and the endocannabinoid system was first evidenced by the massive loss of CB1 receptors in the SN of postmortem HD brains [139]. Subsequent studies in transgenic mouse models confirmed progressive neuronal CB1 receptor loss as a hallmark of HD pathology [140,141,142]. The observed loss of CB1 receptors coupled with significantly reduced AEA levels in the striatum of an HD rat model impaired endocannabinoid transmission, affecting both receptors and their endogenous ligands [143]. In contrast to the early downregulation of CB1 receptors in striatal neurons, upregulation of CB2 receptors has been observed in the striatum of R6/1 and R6/2 transgenic mice, as well as in human HD brains. These CB2 receptors are expressed both in activated M1 microglia and astrocytes. A study by Benito et al. [144] demonstrated that constitutive CB2 receptor activity exerted protective effects against HD progression in R6/2 mice. This was evidenced by reduced proportions of pro-inflammatory M1 microglia and lower levels of inflammatory mediators (IL-1β, IL-6, TNFα, and iNOS) in the striatum, suggesting that CB2 activation helps to mitigate neuroinflammation in this mouse model [144]. Nevertheless, another study reported CB2 receptor upregulation in the vasculature of human HD brains, without localization in microglia or astrocytes [145]. These discrepancies can be explained by different model systems. R6/2 mice, which express human mutant huntingtin exon 1, are only a model of human HD, and their results could differ from those for material from patients.

The endocannabinoid system plays a crucial role in modulating excitotoxicity (via CB1 receptors) and neuroinflammation (via CB2 receptors), making pharmacological strategies targeting these receptors particularly relevant for HD therapy. In cellular HD models, the selective CB1 receptor agonist arachidonyl-2′-chloroethylamide (ACEA) was shown to upregulate neuronal CB1 receptor mRNA and protein expression via NF-dB and Akt downstream of CB1 receptor activation [146]. However, ACEA did not improve the survival of striatal projection neurons in malonate-lesioned Sprague Dawley rats [147]. Similarly, no protective effects of Δ9-THC or HU-210, a synthetic nonselective agonist on CB1 receptor preservation, were observed in the R6/1 HD mice [148]. In contrast, the application of positive allosteric modulators enhanced CB1 receptor activation by endogenous cannabinoids and improved cell viability in a cellular HD model, as well as enhancing motor coordination in R6/2 mice [149]. The inhibition of FAAH has also been shown to maintain CB1 receptors in the striatum of R6/1 mice [145]. Although therapeutic approaches targeting microglial CB2 receptors remain limited, a neuroprotective effect of the CB2-selective agonist HU-308 has been reported in rats [147]. A recently developed strategy targeting microglial CB2 receptors to dampen the neuroinflammatory response appears promising for the treatment of brain diseases with an inflammatory component [150].

### 5.5. Multiple Sclerosis

Both natural and synthetic cannabinoids have been used in MS therapy for years. Clinical observations indicate that MS patients using marijuana experience fewer disease relapses [151]. Experimental MS models demonstrate that the stimulation of CB1 and CB2 receptors can attenuate the inflammatory process [152,153]. Similar anti-inflammatory effects have also been observed in experimental MS models in response to pharmacological α-methyltryptamine (AMT) inhibitors, which increase AEA levels [154,155]. Additionally, studies in a mouse MS model infected with Theiler’s murine encephalomyelitis virus (TMEV) demonstrated that cannabinoids such as WIN 55,212-2, ACEA, and JWH-015 stimulated remyelination while reducing spinal cord infiltration of CD4+ T cells [152,156], and administering the cannabinoid agonist WIN 55,212-2 after viral infection inhibited the expression of adhesion molecules such as intercellular adhesion molecule-1 (ICAM-1) and vascular cell adhesion molecule-1 (VCAM-1). This effect was mediated by the activation of nuclear receptors inhibiting PPARγ, which was accompanied by decreased perivascular infiltration of CD4+ T lymphocytes and attenuated microglial activation. However, contrasting findings from Correa et al. [157] revealed that CB1 and CB2 receptor activation upregulated cyclooxygenase-2 (COX-2) and prostaglandin E2 (PGE2) expression. Additional in vitro studies have demonstrated that AEA plays a key role in neuroinflammatory processes by modulating VCAM-1 expression in brain endothelial cells through CB1 receptor activation. The absence of CB1 receptors exacerbates neuroinflammation, highlighting their protective role in limiting leukocyte migration across the blood–brain barrier (BBB)—a critical step in MS pathogenesis [158]. In a well-characterized TMEV-induced murine MS model, increased expression of the chemokines CCL2 and CCL5 was observed in the spinal cord of infected mice. Treatment with CBD reduced the expression of these chemokines, which correlated with decreased leukocyte infiltration in the brain [159]. Moreover, Guaza et al. [160] showed that using WIN 55,212-2 in mice restored tolerance to a myelin self-antigen, leading to long-term disease amelioration. The authors demonstrated that this effect was associated with decreased activation of CD4+CD25+Foxp3−T cells in the CNS. Additionally, the use of cannabinoids such as THC and CBD in MS treatment may suppress Th17—which is frequently elevated in patients with inflammatory autoimmune disorders like MS—by reducing the synthesis and secretion of IL-17 [161,162].

### 5.6. HIV-Associated Disease

Cannabinoids are believed to mitigate the progression of HIV-associated dementia due to their ability to modulate microglia activation [163]. However, the absence of reliable animal models of HIV-related neuropathology precludes verification of this hypothesis. CB2 receptor activation has been suggested as a potential therapeutic strategy for HIV patients, as it may inhibit viral replication, regulate inflammation by reducing blood–brain barrier permeability and leukocyte infiltration, and suppress the activity of neurotoxic proteins [164].

In clinical trials investigating the anti-inflammatory effects of cannabinoids in neurodegenerative disorders, selecting the right biomarkers is crucial for evaluating efficacy and mechanistic outcomes. Key biomarkers suitable for monitoring these effects could be pro-inflammatory cytokines such as TNF-α, IL-1β, IL-6, are IFN-γ, which are often elevated in neurodegenerative diseases and can be tracked to assess the anti-inflammatory response to cannabinoid therapy. Monitoring increases in anti-inflammatory cytokines like IL-10 and TGF-β may reflect a positive therapeutic response. It also seems reasonable to define the level of acute-phase proteins and inflammatory markers such as CRP—a general systemic inflammation marker—serum amyloid A, and ferritin, which is elevated in some neurodegenerative and inflammatory states. Finally, there are a number of relevant microglial activation markers: sTREM2 (soluble TREM2), which is associated with microglial activation in AD; YKL-40 (Chitinase-3-like protein 1), which is elevated in various neurodegenerative diseases; Iba1 (ionized calcium-binding adapter molecule 1), which is detectable in CSF; and TSPO (Translocator Protein), which is used in PET imaging to measure microglial activation [165].

## 6. The Therapeutic Potential of FAAH Inhibitors in CNS Disorders

Many studies indicate that FAAH could be a therapeutic target for neurological disorders. FAAH is an integral membrane enzyme that is responsible for hydrolyzing endocannabinoid anandamide (AEA) and related amidated signaling lipids. FAAH could be a potential therapeutic target across multiple neurodegenerative diseases due to its role in modulating endocannabinoid levels and its involvement in neuroinflammation and neuroprotection. Blocking or inactivating FAAH induces analgesic, anti-inflammatory, anxiolytic, and antidepressant phenotypes without the side effects commonly associated with direct cannabinoid receptor agonists. FAAH inhibitors enhance the action of AEA and other fatty acid amides, providing a functionally selective approach to increasing endocannabinoid tone exclusively in tissues and cells that actively synthesize and release endocannabinoids [166]. Increased expression of FAAH in the brain has been correlated with reduced levels of lipid amides and exacerbated AD-related symptoms. FAAH levels are increased in hypertrophied astrocytes around amyloid plaques in AD samples, correlating with increased enzymatic activity in plaque areas. This suggests that FAAH may contribute to neuroinflammation in AD [108]. Consequently, FAAH inhibition shows promising potential for alleviating symptoms associated with AD [167]. FAAH inhibitors exert therapeutic effects in neurodegenerative disorders by reducing the levels of cytokines, ROS, and prostaglandins [153]. For example, FAAH inhibitors such as PF-3845 and URB597, in combination with small interfering RNA (siRNA) knockdown, demonstrate neuroprotective properties in BV2 microglial cells by decreasing LPS-induced PGE2 production through downregulation of COX-2 and microsomal PGE synthase [168]. Similarly, URB597 suppresses inflammatory responses in activated microglia by inhibiting COX-2 and iNOS expression, thereby reducing PGE2 and NO release [169]. In PD, FAAH inhibition with URB597 has been shown to increase AEA levels in the brain and improve motor behavior in PD models, but lacks neuroprotective activity. Symptomatic relief is mediated by CB1 and CB2 receptors [170]. On the other hand, FAAH levels are elevated in the R6/2 transgenic mouse model of HD, which may contribute to the disease’s pathogenesis [171]. In patients with HD, FAAH activity is reduced, leading to increased endocannabinoid levels in the blood, suggesting a protective mechanism [172]. In ALS models, FAAH inhibition delayed disease progression and improved survival, likely by reducing the levels of pro-inflammatory cytokines and increasing BDNF levels [173]. FAAH inhibitors also modulate transient receptor potential vanilloid 1 (TRPV1) and toll-like receptor 4 (TLR4)-associated neuroinflammation in microglia [174].

## 7. Conclusions

Cannabinoids, the active compounds derived from Cannabis sativa, are attracting increasing interest for their therapeutic potential in neurodegenerative disorders (Parkinson’s disease, Alzheimer’s disease, and Huntington’s disease) and inflammatory CNS conditions (multiple sclerosis and HIV-associated dementia). Their multimodal mechanisms of action include the following: (1) modulating pathological protein aggregation and mitochondrial dysfunction, and (2) exerting neuroprotective and anti-inflammatory effects which are mediated through microglial regulation. The neurodegenerative diseases and inflammatory CNS disorders discussed in this work represent a serious challenge for healthcare systems due to their complex etiology or pathophysiology, severe symptoms, and the limited effectiveness of existing treatments. Consequently, improving therapeutic strategies for these disorders remains a priority. Many studies suggest that pharmacological modulation of the endocannabinoid system could influence neurodegenerative processes, providing a basis for further research into cannabinoid-based therapies. In particular, the inhibition of FAAH in the endocannabinoid system has emerged as a potential therapeutic approach to control neuroinflammatory processes. While these findings are encouraging, the translation of cannabinoid-based therapies into clinical practice requires further rigorous investigation. Key priorities include establishing optimal dosing regimens, evaluating long-term safety profiles, and conducting large-scale clinical trials to validate efficacy in different disease stages and patient populations. Chronic use of cannabinoids in patients with neurodegenerative diseases can offer therapeutic benefits, but it also comes with potential side effects and long-term risks. These vary depending on the type of cannabinoid, dosage, frequency of use, and individual patient factors (e.g., age, other medications, stage of disease). Potential side effects include cognitive effects, such as memory impairment, slower reaction time, and reduced attention or executive function, as well as psychiatric effects, like anxiety or paranoia (especially with THC), mood swings, and, in rare cases, hallucinations, especially in older adults or those with a predisposition to psychosis [175]. Therapy with cannabinoids could also cause gastrointestinal problems like nausea, diarrhea, and appetite changes, as well as some cardiovascular effects, such as increased heart rate and changes in blood pressure. Its long-term risks include tolerance and dependence, as well as mental health risks: long-term use of THC is known to be linked with a heightened risk of depression and anxiety in some cases. The use of non-psychotropic cannabinoids, such as cannabidiol, or the development of allosteric modulators targeting cannabinoid receptors present promising therapeutic strategies for neurodegenerative diseases. Unlike direct agonists of CB1 receptors, which often produce undesirable psychotropic effects, these alternatives can modulate the endocannabinoid system more selectively and safely. Allosteric modulators, in particular, can fine-tune receptor activity without directly activating the receptors, potentially enhancing therapeutic outcomes while minimizing side effects. This approach may allow for the beneficial regulation of neuroinflammation and neurodegeneration without compromising cognitive or behavioral function.

## Data Availability

Not applicable.

## Abbreviations

2-AG	2-arachidonylglycerol
Aβ	amyloid plaques
AD	Alzheimer’s disease
AEA	anandamide
ALS	amyotrphic lateral sclerosis
AJs	adherens junctions
BBB	blood–brain barrier
BDNF	brain-derived neurotrophic factor
CB	cannabinoid
CB1	cannabinoid receptor 1
CB2	cannabinoid receptor 2
CBD	cannabidiol
CBRs	cannabinoid receptors
CNS	Central Nervous System
COX	cyclooxygenase
CSF	cerebrospinal fluid
CX3-CX3R1	CX3 motif receptor 1
ECB	endocannabinoids
FAAH	fatty acid amide hydrolase
FasL	fas lipase
gp130	glycoprotein 130
GPR55	G protein-coupled receptor 55
HD	Huntington’s disease
IFNγ	interferon gamma
IL	interleukin
IL-1Ra	interleukin—1 receptor antagonist
JAK/STAT	Janus kinases/signal transducer and activator of transcription
LPS	lipopolysaccharide
MAPK	mitogen activated protein kinase
MHC	major histocompatibility complex
NFkΒ	nuclear factor kappa Β
NFTs	neurofibrillary tangles
NO	nitric oxide
NSAID	non-steroidal anti-inflammatory drugs
OPCs	oligodendrocyte progenitor cells
PD	Parkinson’s disease
PPARγ	peroxisome proliferator-activated receptor γ
ROS	reactive oxygen species
SN	substantia nigra
SOCS3	suppressor of cytokine signaling 3
SOD	superoxide dismutase
STAT3	signal transductor and activator of transcription 3
TJs	tight junctions
TGFβ	transforming growth factor beta
TNF-α	tumor necrosis factor α
TRKβ	tyrosine receptor kinase β
TRPV1	transient receptor potential cation channel subfamily V member 1
VEGF	vascular endothelial growth factor

## References

[1] Finazzi-Agrò A., Maccarrone M., Bernardi G., Centonze D. (2007). The endocannabinoid system in targeting inflammatory neurodegenerative diseases. Trends Pharmacol. Sci..

[2] Fowler C.J. (2005). Pharmacological Properties and Therapeutic Possibilities for Drugs Acting Upon Endocannabinoid Receptors. CNS Neurol. Disord. Drug Targets.

[3] Sharma N., Singh S., Uivarosan D., Makkar R., Zengin G., Brisc M.C., Sehgal A., Andronie-Cioara F.L., Bungau S., Munteanu M.A. (2021). Current Trends in Neurodegeneration: Cross Talks between Oxidative Stress, Cell Death, and Inflammation. Int. J. Mol. Sci..

[4] Knight J., Caseldine C., Boykoff M.T. (2010). Forum review. Geogr. J..

[5] Colombo E., Farina C. (2016). Astrocytes: Key Regulators of Neuroinflammation. Trends Immunol..

[6] Porazzi P., Boehm A., Xiao D., Ciric B., Li X., Tang H.-Y., Ishikawa L.L.W., Rasouli J., Hwang D., Thome R. (2020). Oligodendrocyte-derived extracellular vesicles as antigen-specific therapy for autoimmune neuroinflammation in mice. Sci. Transl. Med..

[7] Ludwin S., Yaqubi M., Antel J.P., Healy L.M. (2019). Species differences in immune-mediated CNS tissue injury and repair: A (neuro)inflammatory topic. Glia.

[8] Pasolli H.A., Hess H.F., Matthies D., Pang S., Jackson J., Sheu S.-H., Liu Z., Ioannou M.S., Liu H., Chang C.-L. (2019). Neuron-Astrocyte Metabolic Coupling Protects against Activity-Induced Fatty Acid Toxicity. Cell.

[9] Latz E., McManus R.M., Heneka M.T. (2018). Inflammasome signalling in brain function and neurodegenerative disease. Nat. Rev. Neurosci..

[10] Ffrench-Constant C., Samudyata, Williams A., Guerreiro-Cacais A.O., Floriddia E.M., Agirre E., van Bruggen D., Marques S., Castelo-Branco G., Meijer M. (2018). Disease-specific oligodendrocyte lineage cells arise in multiple sclerosis. Nat. Med..

[11] Lelios I., Croxford A.L., Greter M. (2015). Microglia Versus Myeloid Cell Nomenclature during Brain Inflammation. Front. Immunol..

[12] Nimmerjahn A., Kirchhoff F., Helmchen F. (2005). Resting Microglial Cells Are Highly Dynamic Surveillants of Brain Parenchyma in Vivo. Science.

[13] Hanamsagar R., Hanke M.L., Kielian T. (2012). Toll-like receptor (TLR) and inflammasome actions in the central nervous system. Trends Immunol..

[14] Schmelzer L., Guttridge D.C., Ladner K.J., Godbout J.P., Popovich P.G., Foust K.D., Miranda C.J., Kaspar B.K., Bevan A.K., Haidet-Phillips A.M. (2014). Microglia Induce Motor Neuron Death via the Classical NF-κB Pathway in Amyotrophic Lateral Sclerosis. Neuron.

[15] Van Eldik L.J., Smith C.J., Kim S.H. (2004). Importance of MAPK pathways for microglial pro-inflammatory cytokine IL-1β production. Neurobiol. Aging.

[16] Verma K., Paliwal S., Bisht A., Negi S., Jain S., Sharma S. (2021). The role of fatty acid amide hydrolase enzyme inhibitors in Alzheimer’s disease. Cell Biochem. Funct..

[17] Cardona A.E., Pioro E.P., Sasse M.E., Kostenko V., Cardona S.M., Dijkstra I.M., Huang D., Kidd G., Dombrowski S., Dutta R. (2006). Control of microglial neurotoxicity by the fractalkine receptor. Nat. Neurosci..

[18] Wu G., Ren S., Hang H., Wang L. (2020). Up-regulation of PPARγ, Nrf2 and HO-1 in microglia activated by thrombin. Brain Hemorrhages.

[19] Sarkar S. (2022). Microglial ion channels: Key players in non-cell autonomous neurodegeneration. Neurobiol. Dis..

[20] Hanisch U.K., Kettenmann H. (2007). Microglia: Active sensor and versatile effector cells in the normal and pathologic brain. Nat. Neurosci..

[21] Weiner H.L., Butovsky O. (2018). Microglial signatures and their role in health and disease. Nat. Rev. Neurosci..

[22] Gabellini C., Piano I., Cerri C., Puppi D., Leigheb M., Votta A., Colucci P., Maya-Vetencourt J.F., Lai M., Corsi F. (2023). Anti-inflammatory reprogramming of microglia cells by metabolic modulators to counteract neurodegeneration; a new role for Ranolazine. Sci. Rep..

[23] Olschowka J.A., Cherry J.D., O’Banion M.K. (2014). Are “Resting” Microglia More “M2”?. Front. Immunol..

[24] Degos V., Chhor V., Le Charpentier T., Lebon S., Oré M.-V., Celador I.L., Josserand J., Sävman K., Hagberg H., Mallard C. (2013). Characterization of phenotype markers and neuronotoxic potential of polarised primary microglia in vitro. Brain Behav. Immun..

[25] Deierborg T., Yang Y., Paulus A., Jiménez-Ferrer I., Swanberg M., Bachiller S., Boza-Serrano A. (2018). Microglia in Neurological Diseases: A Road Map to Brain-Disease Dependent-Inflammatory Response. Front. Cell. Neurosci..

[26] Gordon S., Varin A. (2009). Alternative activation of macrophages: Immune function and cellular biology. Immunobiology.

[27] Cherry J.D., Olschowka J.A., O’Banion M.K. (2014). Neuroinflammation and M2 microglia: The good, the bad, and the inflamed. J. Neuroinflamm..

[28] Gabriely G., Gygi S.P., Jedrychowski M.P., Dake B., Chen Z., Rothstein J.D., Moore C.S., Koeglsperger T., Weiner H.L., Butovsky O. (2013). Identification of a unique TGF-β–dependent molecular and functional signature in microglia. Nat. Neurosci..

[29] Prinz M., Staszewski O., Sankowski R., Masuda T. (2020). Microglia Heterogeneity in the Single-Cell Era. Cell Rep..

[30] Gosselin D., Skola D., Coufal N.G., Holtman I.R., Schlachetzki J.C.M., Sajti E., Jaeger B.N., O’Connor C., Fitzpatrick C., Pasillas M.P. (2017). An environment-dependent transcriptional network specifies human microglia identity. Science.

[31] Sortino M.A., Gullotta G.S., Costantino G., Spampinato S.F. (2023). Microglia and the Blood–Brain Barrier: An External Player in Acute and Chronic Neuroinflammatory Conditions. Int. J. Mol. Sci..

[32] Iadecola C. (2017). The Neurovascular Unit Coming of Age: A Journey through Neurovascular Coupling in Health and Disease. Neuron.

[33] Daneman R., Prat A. (2015). The blood-brain barrier. Cold Spring Harb. Perspect. Biol..

[34] Tietz S., Engelhardt B. (2015). Brain barriers: Crosstalk between complex tight junctions and adherens junctions. J. Cell Biol..

[35] Hagan K., Varelas P., Zheng H.A. (2022). Endocannabinoid system of the blood-brain B\barrier: Current understandings and therapeutic potentials. Cannabis. Cannabinoid. Res..

[36] Lu T.-S., Avraham H.K., Seng S., Tachado S.D., Koziel K., Makriyannis A., Avraham S. (2008). Cannabinoids inhibit HIV-1 Gp120-mediated insults in brain microvascular endothelial cells. J. Immunol..

[37] Amenta P.S., Jallo J.I., Tuma R.F., Hooper D.C., Elliott M.B. (2014). Cannabinoid receptor type-2 stimulation, blockade, and deletion alter the vascular inflammatory responses to traumatic brain injury. J. Neuroinflammation.

[38] Dudek K.A., Paton S.E.J., Binde L.B., Collignon A., Dion-Albert L., Cadoret A., Lebel M., Lavoie O., Bouchard J., Kaufmann F.N. (2025). Astrocytic cannabinoid receptor 1 promotes resilience by dampening stress-induced blood-brain barrier alterations. Nat. Neurosci..

[39] Penna E., Tarantal A.F., Cunningham C.L., Saylor S., Martínez-Cerdeño V., Kreutz A., Noctor S.C. (2021). Greater Number of Microglia in Telencephalic Proliferative Zones of Human and Nonhuman Primate Compared with Other Vertebrate Species. Cereb. Cortex Commun..

[40] Greferath U., Jobling A.I., Dixon M.A., Fletcher E.L. (2021). The Contribution of Microglia to the Development and Maturation of the Visual System. Front. Cell. Neurosci..

[41] Sun Y.-Y., Kuan C.-Y., Mills W.A., Jabbour L., Bisht K., Campos-Salazar A.B., Amancherla S., Calcuttawala Z., Benderoth J., Friestad B. (2021). Capillary-associated microglia regulate vascular structure and function through PANX1-P2RY12 coupling in mice. Nat. Commun..

[42] Morales I., Farías G., Navarrete L., Maccioni R.B. (2010). The Revitalized Tau Hypothesis on Alzheimer’s Disease. Arch. Med. Res..

[43] Heneka M.T., Klockgether T., Sastre M. (2006). Contribution of inflammatory processes to Alzheimer’s disease: Molecular mechanisms. Int. J. Dev. Neurosci..

[44] Lebeurrier N., Cacquevel M., Vivien D., Cheenne S. (2004). Cytokines in Neuroinflammation and Alzheimers Disease. Curr. Drug Targets.

[45] Akiyama H. (2000). Inflammation and Alzheimer’s disease. Neurobiol. Aging.

[46] Marchetti B., Abbracchio M.P. (2005). To be or not to be (inflamed)—Is that the question in anti-inflammatory drug therapy of neurodegenerative disorders?. Trends Pharmacol. Sci..

[47] Keren-Shaul H., Spinrad A., Weiner A., Matcovitch-Natan O., Dvir-Szternfeld R., Ulland T.K., David E., Baruch K., Lara-Astaiso D., Toth B. (2017). A Unique Microglia Type Associated with Restrict-ing Development of Alzheimer’s Disease. Cell.

[48] Zandi P.P., Breitner J.C., Mayer L., Mehta K., Anthony J.C., Hayden K.M. (2002). Reduced incidence of AD with NSAID but not H_2_ receptor antagonists. Neurology.

[49] McGeer P.L., McGeer E.G. (2004). Inflammation and neurodegeneration in Parkinson’s disease. Park. Relat. Disord..

[50] Gao H.-M., Zhang W., Liu B., Hong J.-S. (2003). Novel anti-inflammatory therapy for Parkinson’s disease. Trends Pharmacol. Sci..

[51] Del Dotto P., Bonuccelli U. (2006). New pharmacologic horizons in the treatment of Parkinson disease. Neurology.

[52] Block M.L., Hong J.-S. (2005). Microglia and inflammation-mediated neurodegeneration: Multiple triggers with a common mechanism. Prog. Neurobiol..

[53] Nappi G., Bazzini E., Levandis G., Armentero M.-T., Blandini F. (2006). Peripheral inflammation and neuroprotection: Systemic pretreatment with complete Freund’s adjuvant reduces 6-hydroxydopamine toxicity in a rodent model of Parkinson’s disease. Neurobiol. Dis..

[54] Rahim F., Rabbani Z., Aghayan H.R., Tayanloo-Beik A., Hamidpour S.K., Arjmand B., Larijani B. (2022). Organ on a Chip: A Novel in vitro Biomimetic Strategy in Amyotrophic Lateral Sclerosis (ALS) Modeling. Front. Neurol..

[55] Appel S.H., Alexianu M.E., Kozovska M. (2001). Immune reactivity in a mouse model of familial ALS correlates with disease progression. Neurology.

[56] Andersen P.M., Marklund S.L., Oja S.S., Koistinaho J., Vartiainen N.E., Tikka T.M., Goldsteins G. (2002). Minocycline prevents neurotoxicity induced by cerebrospinal fluid from patients with motor neurone disease. Brain.

[57] Qin W., Ho L., Pasinetti G.M., Bianchi M., McManus T., Pompl P.N. (2003). A therapeutic role for cyclooxygenase-2 inhibitors in a transgenic mouse model of amyotrophic lateral sclerosis. FASEB J..

[58] Gordon P.H., Doorish C., Montes J., Mosley R.L., Diamond B., Macarthur R.B., Weimer L.H., Kauf-mann P., Hays A.P., Rowland L.P. (2006). Randomized con-trolled phase II trial of glatiramer acetate in ALS. Neurology.

[59] Linker R.A., Reick C., Saft C., Ellrichmann G. (2013). The Role of the Immune System in Huntington’s Disease. J. Immunol. Res..

[60] Di Marzo V., Cristino L., Bisogno T. (2019). Cannabinoids and the expanded endocannabinoid system in neurological disorders. Nat. Rev. Neurol..

[61] Hashikawa T., Aronin N., Sapp E., Vonsattel J.P., Uchiyama Y., Tohyama K., Kegel K.B., Difiglia M., Bhide P.G. (2001). Early and Progressive Accumulation of Reactive Microglia in the Huntington Disease Brain. J. Neuropathol. Exp. Neurol..

[62] Pavese N., Gerhard A., Tai Y.F., Ho A.K., Turkheimer F., Barker R.A., Brooks D.J., Piccini P. (2006). Microglial activation correlates with severity in Huntington disease: A clinical and PET study. Neurology.

[63] Tai Y.F., Pavese N., Gerhard A., Tabrizi S.J., Barker R.A., Brooks D.J., Piccini P. (2007). Microglial activation in presymptomatic Huntington’s disease gene carriers. Brain.

[64] Brundin P., Magnusson A., Lowdell M.W., Silvestroni A., Woodman B., Benn C.L., Andre R., Lee R.V., Khalili-Shirazi A., Raibon E. (2008). A novel pathogenic pathway of immune activation detectable before clinical onset in Huntington’s disease. J. Exp. Med..

[65] Andrich J., Przuntek H., Zange J., Lindenberg K., Kraus P.H., Vorgerd M., Schöls L., Müller K., Landwehrmeyer B., Saft C. (2005). Mitochondrial impairment in patients and asymptomatic mutation carriers of Huntington’s disease. Mov. Disord..

[66] Kipnis J., Schwartz M. (2005). Protective autoimmunity and neuroprotection in inflammatory and noninflammatory neurodegenerative diseases. J. Neurol. Sci..

[67] Selter R.C., Hemmer B. (2013). Update on immunopathogenesis and immunotherapy in multiple sclerosis. ImmunoTargets Ther..

[68] Trapp B.D., Rudick R., Bö L., Mörk S., Ransohoff R.M., Peterson J. (1998). Axonal Transection in the Lesions of Multiple Sclerosis. N. Engl. J. Med..

[69] Oksenberg J.R., Hauser S.L. (2006). The Neurobiology of Multiple Sclerosis: Genes, Inflammation, and Neurodegeneration. Neuron.

[70] Masuda T., Sankowski R., Staszewski O., Bottcher C., Amann L., Sagar, Scheiwe C., Nessler S., Kunz P., van Loo G. (2019). Spatial and temporal heterogeneity of mouse and human microglia at single-cell reso-lution. Nature.

[71] Di Tommaso M., Konje J.C., Pirazzi V., Maccarrone M., Battista N., Fasano S., Pierantoni R., Cobellis G., Meccariello R. (2012). The role of endocannabinoids in gonadal function and fertility along the evolutionary axis. Mol. Cell. Endocrinol..

[72] Di Marzo V., Schinelli S., Piomelli D., Cadas H., Cimino G., Schwartz J.-C., Fontana A. (1994). Formation and inactivation of endogenous cannabinoid anandamide in central neurons. Nature.

[73] Hunyady L., Turu G. (2009). Signal transduction of the CB1 cannabinoid receptor. J. Mol. Endocrinol..

[74] Zou S., Kumar U. (2018). Cannabinoid Receptors and the Endocannabinoid System: Signaling and Function in the Central Nervous System. Int. J. Mol. Sci..

[75] Tanasescu R., Constantinescu C.S. (2010). Cannabinoids and the immune system: An overview. Immunobiology.

[76] Parolaro D., Massi P., Vaccani A. (2006). Cannabinoids, Immune System and Cytokine Network. Curr. Pharm. Des..

[77] Orlando P., Pochard P., Matias I., Pestel J., Salzet M., Di Marzo V. (2002). Presence and regulation of the endocannabinoid system in human dendritic cells. Eur. J. Biochem..

[78] Carrier E.J., Hillard C.J., Nithipatikom K., Yang W., Pfister S.L., Kearn C.S., Barkmeier A.J., Breese N.M., Campbell W.B. (2004). Cultured Rat Microglial Cells Synthesize the Endocannabinoid 2-Arachidonylglycerol, Which Increases Proliferation via a CB2 Receptor-Dependent Mechanism. Mol. Pharmacol..

[79] Yamamura T., Croxford J.L. (2005). Cannabinoids and the immune system: Potential for the treatment of inflammatory diseases?. J. Neuroimmunol..

[80] Tanasescu R., Gran B., Constantinescu C.S. (2012). The endocannabinoid system: A revolving plate in neuro-immune interaction in health and disease. Amino Acids.

[81] Newton C., Nong L., Friedman H., Perkins I., Larsen K., Lu L., Klein T.W. (2003). The cannabinoid system and immune modulation. J. Leukoc. Biol..

[82] Denovan-Wright E.M., Young A.P. (2022). Synthetic cannabinoids reduce the inflammatory activity of microglia and subsequently improve neuronal survival in vitro. Brain Behav. Immun..

[83] Almogi-Hazan O., Or R. (2020). *Cannabis*, the Endocannabinoid System and Immunity—The Journey from the Bedside to the Bench and Back. Int. J. Mol. Sci..

[84] Lefebvre J.S., Chouinard F., Turcotte C., Flamand N. (2015). Regulation of inflammation by cannabinoids, the endocannabinoids 2-arachidonoyl-glycerol and arachidonoyl-ethanolamide, and their metabolites. J. Leukoc. Biol..

[85] Stella N. (2009). Endocannabinoid signaling in microglial cells. Neuropharmacology.

[86] Stella N. (2010). Cannabinoid and cannabinoid-like receptors in microglia, astrocytes, and astrocytomas. Glia.

[87] Pandey R., Mousawy K., Nagarkatti M., Nagarkatti P. (2009). Endocannabinoids and immune regulation. Pharmacol. Res..

[88] Mecha M., Guaza C., Mestre L., Feliú A., Carrillo-Salinas F. (2016). Microglia activation states and cannabinoid system: Therapeutic implications. Pharmacol. Ther..

[89] Duffy S.S., Fiore N.T., Moalem-Taylor G., Hayes J.P. (2021). The cannabinoid system and microglia in health and disease. Neuropharmacology.

[90] Dvir-Ginzberg M., Lutz B., Palmisano M., Bilkei-Gorzo A., de Almodovar C.R., Farhat E., Ramunno C.F. (2024). Local cannabinoid receptor type-1 regulates glial cell activity and insulin-like growth factor-1 receptor signaling in the mediobasal hypothalamus. Mech. Ageing Dev..

[91] Fuxe K., Lanciego J.L., Saura C.A., Pulido-Salgado M., Labandeira-García J.L., Reyes-Resina I., Borroto-Escuela D., Rodríguez-Pérez A.I., Canela E.I., Saura J. (2018). Receptor-heteromer mediated regulation of endocannabinoid signaling in activated microglia. Role of CB1 and CB2 receptors and relevance for Alzheimer’s disease and levodopa-induced dyskinesia. Brain Behav. Immun..

[92] Guaza C., Mecha M., Rueda-Zubiaurre A., de Sola R.G., Feliú A., Carrillo-Salinas F., Ortega-Gutiérrez S. (2015). Endocannabinoids drive the acquisition of an alternative phenotype in microglia. Brain Behav. Immun..

[93] Hu S., Cabral G.A., Peterson P.K., Sheng W.S., Min X., Lokensgard J.R. (2004). Synthetic cannabinoid WIN55,212-2 inhibits generation of inflammatory mediators by IL-1β-stimulated human astrocytes. Glia.

[94] Sáez J.C., Ezan P., Froger N., Amigou E., Giaume C., Orellana J.A., Cohen-Salmon M. (2009). Cannabinoids prevent the opposite regulation of astroglial connexin43 hemichannels and gap junction channels induced by pro-inflammatory treatments. J. Neurochem..

[95] Marsicano G., Pouvreau S., Skupio U., Gomez-Sotres P., Olivera-Pinto A., Eraso-Pichot A. (2022). Endocannabinoid signaling in astrocytes. Glia.

[96] Tomczyk M., Herman A., Tomaszewska-Zaremba D., Herman A.P., Bochenek J. (2021). Anandamide Influences Interleukin-1β Synthesis and *IL-1* System Gene Expressions in the Ovine Hypothalamus during Endo-Toxin-Induced Inflammation. Animals.

[97] Lutz B., Bilkei-Gorzo A., Berger M., Zimmer A., Leidmaa E., Nidadavolu P., Schürmann B., Bindila L., Bailey A., Effah F. (2022). Dynamic Changes in the Endocannabinoid System during the Aging Process: Focus on the Middle-Age Crisis. Int. J. Mol. Sci..

[98] Young A.P., Denovan-Wright E.M. (2022). The dynamic Role of microglia and the endocannabinoid system in neuroinflammation. Front. Pharmacol..

[99] Leonard B.E., Aricioglu F. (2023). Cannabinoids and neuroinflammation: Therapeutic implications. J. Affect. Disord. Rep..

[100] Sadanandan S.M., Kreko-Pierce T., Khatri S.N., Pugh J.R. (2020). Cannabinoid type 2 receptors inhibit GABAA receptor-mediated currents in cerebellar Purkinje cells of juvenile mice. PLoS ONE.

[101] Zhang H.Y., Shen H., Gao M., Ma Z., Hempel B.J., Bi G.H., Gardner E.L., Wu J., Xi Z.X. (2021). Cannabinoid CB2 receptors are expressed in glutamate neurons in the red nucleus and functionally modulate motor behavior in mice. Neuropharmacology.

[102] Jîtcă G., Ősz B.E., Vari C.E., Rusz C.-M., Tero-Vescan A., Pușcaș A. (2023). Cannabidiol: Bridge between Antioxidant Effect, Cellular Protection, and Cognitive and Physical Performance. Antioxidants.

[103] Yu L., Li S., Huang Y., Ji X., Wu J. (2023). Impact of the Cannabinoid System in Alzheimer’s Disease. Curr. Neuropharmacol..

[104] Blázquez C., Ramírez B.G., del Pulgar T.G., Guzmán M., de Ceballos M.L. (2005). Prevention of Alzheimer’s Disease Pathology by Cannabinoids: Neuroprotection Mediated by Blockade of Microglial Activation. J. Neurosci..

[105] Matias I., Petrosino S., De Filippis D., Mazzola C., Iuvone T., Micale V., van der Stelt M., Di Marzo V., Esposito G., Drago F. (2006). Endocannabinoids and β-amyloid-induced neurotoxicity in vivo: Effect of pharmacological elevation of endocannabinoid levels. Cell. Mol. Life Sci..

[106] Milton N.G. (2002). Anandamide and noladin ether prevent neurotoxicity of the human amyloid-β peptide. Neurosci. Lett..

[107] Tanila H., Alpár A., Pasquaré S.J., Martín-Moreno A.M., Watanabe M., Schulte G., Mackie K., Mulder J., Zilberter M., Keimpema E. (2011). Molecular reorganization of endocannabinoid signalling in Alzheimer’s disease. Brain.

[108] Benito C., Tolón R.M., Carrier E.J., Hillard C.J., Núñez E., Rábano A., Romero J. (2003). Cannabinoid CB_2_ Receptors and Fatty Acid Amide Hydrolase Are Selectively Overexpressed in Neuritic Plaque-Associated Glia in Alzheimer’s Disease Brains. J. Neurosci..

[109] Halleskog C., Dahlström J., Tanila H., Färber K., Harkany T., Mackie K., Mulder J., Schulte G., Puli L.K., Hortobágyi T. (2010). WNT signaling in activated microglia is proinflammatory. Glia.

[110] Head E., Yasar S., Piomelli D., Cribbs D.H., Vasilevko V., Cotman C.W., Jung K.-M., Astarita G. (2012). An amyloid β42-dependent deficit in anandamide mobilization is associated with cognitive dysfunction in Alzheimer’s disease. Neurobiol. Aging.

[111] Calcagnini S., De Marco F., Gaetani S., Pace L., Cassano T., Romano A. (2017). Cannabinoid Receptor 2 Signaling in Neurodegenerative Disorders: From Pathogenesis to a Promising Therapeutic Target. Front. Neurosci..

[112] Li C., Wang B., Li J., Shi J., Jia H. (2019). CB2 cannabinoid receptor agonist ameliorates novel object recognition but not spatial memory in transgenic APP/PS1 mice. Neurosci. Lett..

[113] Benito C., Tolón R.M., Castillo A.I., Romero J., Núñez E., Pazos M.R., Martínez-Orgado J.A. (2009). The activation of cannabinoid CB2 receptors stimulates in situ and in vitro beta-amyloid removal by human macrophages. Brain Res..

[114] Maldonado R., Juvés S., Ferrer I., Aso E. (2013). CB2 Cannabinoid Receptor Agonist Ameliorates Alzheimer-Like Phenotype in AβPP/PS1 Mice. J. Alzheimer’s Dis..

[115] Monteiro K.L.C., de Aquino T.M., da Silva-Júnior E.F., Alcântara M.G.d.S. (2021). Cannabinoid pharmacology and its therapeutic uses in Alzheimer’s disease. Neural Regen. Res..

[116] Ruthirakuhan M., Herrmann N., Gallagher D., Verhoeff N.P.L., Kiss A., Black S.E., Lanctôt K.L. (2019). Randomized Placebo-Controlled Trial of Nabilone for Agitation in Alzheimer’s Disease. Am. J. Geriatr. Psychiatry.

[117] Russo E.B. (2018). Cannabis Therapeutics and the Future of Neurology. Front. Integr. Neurosci..

[118] Patel V., Park O., Bátkai S., Haskó G., Liaudet L., Tanchian G., Horváth B., Mechoulam R., Wink D.A., Pacher P. (2011). Cannabidiol protects against hepatic ischemia/reperfusion injury by attenuating inflammatory signaling and response, oxidative/nitrative stress, and cell death. Free Radic. Biol. Med..

[119] Hamelink C., Hampson A., Wink D.A., Eskay R.L., Eiden L.E. (2005). Comparison of Cannabidiol, Antioxidants, and Diuretics in Reversing Binge Ethanol-Induced Neurotoxicity. J. Pharmacol. Exp. Ther..

[120] McGeer P.L., Itagaki S., Boyes B.E., McGeer E.G. (1988). Reactive microglia are positive for HLA-DR in the substantia nigra of Parkinson’s and Alzheimer’s disease brains. Neurology.

[121] Ouchi Y., Yoshikawa E., Sekine Y., Futatsubashi M., Kanno T., Ogusu T., Torizuka T. (2005). Microglial activation and dopamine terminal loss in early Parkinson’s disease. Ann. Neurol..

[122] Gerhard A., Pavese N., Hotton G., Turkheimer F., Es M., Hammers A., Eggert K., Oertel W., Banati R.B., Brooks D.J. (2006). In vivo imaging of microglial activation with [11C](R)-PK11195 PET in idiopathic Parkinson’s disease. Neurobiol. Dis..

[123] Yoshida M., Hashizume Y., Hishikawa N., Sawada M., Imamura K., Nagatsu T. (2003). Distribution of major histocompatibility complex class II-positive microglia and cytokine profile of Parkinson’s disease brains. Acta Neuropathol..

[124] Lipton J.W., Collier T.J., O McGuire S., Ling Z.D., Carvey P.M., E Sortwell C. (2001). Tumor Necrosis Factor α Is Toxic to Embryonic Mesencephalic Dopamine Neurons. Exp. Neurol..

[125] Aracil-Fernández A., García-Gutiérrez M.S., Lanciego J.L., Navarrete F., Manzanares J. (2018). Cannabinoid CB1 and CB2 Receptors, and Monoacylglycerol Lipase Gene Expression Alterations in the Basal Ganglia of Patients with Parkinson’s Disease. Neurotherapeutics.

[126] Thiolat M.-L., Luquin M.R., Li Q., Bezard E., Clavero P., Rojo-Bustamante E., Abellanas M.A., Aymerich M.S. (2018). The expression of cannabinoid type 1 receptor and 2-arachidonoyl glycerol synthesizing/degrading enzymes is altered in basal ganglia during the active phase of levodopa-induced dyskinesia. Neurobiol. Dis..

[127] Palomo-Garo C., Gómez-Gálvez Y., García C., Fernández-Ruiz J. (2016). Potential of the cannabinoid CB2 receptor as a pharmacological target against inflammation in Parkinson’s disease. Prog. Neuro-Psychopharmacol. Biol. Psychiatry.

[128] Price D.A., Martinez A.A., Seillier A., Koek W., Acosta Y., Fernandez E., Strong R., Lutz B., Marsicano G., Roberts J.L. (2009). WIN55,212-2, a cannabinoid receptor agonist, protects against nigrostriatal cell loss in the 1-methyl-4-phenyl-1,2,3,6-tetrahydropyridine mouse model of Parkinson’s disease. Eur. J. Neurosci..

[129] DI Marzo V. (2000). Enhanced levels of endogenous cannabinoids in the globus pallidus are associated with a reduction in movement in an animal model of Parkinson’s disease. FASEB J..

[130] Mounsey R.B., Mustafa S., Robinson L., Ross R.A., Riedel G., Pertwee R.G., Teismann P. (2015). Increasing levels of the endocannabinoid 2-AG is neuroprotective in the 1-methyl-4-phenyl-1,2,3,6-tetrahydropyridine mouse model of Parkinson’s disease. Exp. Neurol..

[131] Azimullah S., Haque M.E., Ojha S.K., Javed H. (2016). Cannabinoid Type 2 (CB2) Receptors Activation Protects against Oxidative Stress and Neuroinflammation Associated Dopaminergic Neurodegeneration in Rotenone Model of Parkinson’s Disease. Front. Neurosci..

[132] Azimullah S., Haque M.E., Ojha S., Javed H. (2016). β-Caryophyllene, a phytocannabinoid attenuates oxidative stress, neuroinflammation, glial activation, and salvages dopaminergic neurons in a rat model of Parkinson disease. Mol. Cell. Biochem..

[133] Molina-Holgado F., Fernández-Ruiz J., Mechoulam R., Ramos J.A., Lastres-Becker I. (2005). Cannabinoids provide neuroprotection against 6-hydroxydopamine toxicity in vivo and in vitro: Relevance to Parkinson’s disease. Neurobiol. Dis..

[134] Kim S.U., Kim S.R., Jin B.K., Chung E.S., Lee D.Y., Oh U.T. (2005). Transient Receptor Potential Vanilloid Subtype 1 Mediates Cell Death of Mesencephalic Dopaminergic Neurons In Vivo and In Vitro. J. Neurosci..

[135] De Petrocellis L., Bisogno T., Di Marzo V. (2001). Anandamide: Some like it hot. Trends Pharmacol. Sci..

[136] Cravatt B.F., McKinney M.K. (2005). Structure and function of fatty acid amide hydrolase. Annu. Rev. Biochem..

[137] McAllister S.D., Moore D.H., Raman C., Rizvi G., Patel S.G., E Abood M. (2004). Amyotrophic lateral sclerosis: Delayed disease progression in mice by treatment with a cannabinoid. Amyotroph. Lateral Scler..

[138] Petrosino S., Bilsland L.G., Greensmith L., Pryce G., Dick J.R.T., Baker D., Di Marzo V. (2006). Increasing cannabinoid levels by pharmacological and genetic manipulation delays disease progression in SOD1 mice. FASEB J..

[139] Dragunow M., Faull R., Glass M. (1993). Loss of cannabinoid receptors in the substantia nigra in huntington’s disease. Neuroscience.

[140] Møller M., Sørensen S., Stub C., Hasholt L., Fenger K., Hansen A., Naver B. (2003). Molecular and behavioral analysis of the r6/1 huntington′s disease transgenic mouse. Neuroscience.

[141] Bradshaw H., Dowie M., Howard M., Nicholson L., Faull R., Hannan A., Glass M. (2009). Altered CB1 receptor and endocannabinoid levels precede motor symptom onset in a transgenic mouse model of Huntington’s disease. Neuroscience.

[142] Benito C., Julien B., Lutz B., Fernández-Ruiz J., Monory K., Börner C., Ruiz C., Blázquez C., Chiarlone A., Resel E. (2010). Loss of striatal type 1 cannabinoid receptors is a key pathogenic factor in Huntington’s disease. Brain.

[143] Berrendero F., Hansen H.H., De Miguel R., Ramos J.A., Pérez-Rosado A., Lastres-Becker I., Manzanares J., Fernández-Ruiz J. (2002). Alleviation of motor hyperactivity and neurochemical deficits by endocannabinoid uptake inhibition in a rat model of Huntington’s disease. Synapse.

[144] Benito C., Julien B., Fernández-Ruiz J., Resel E., Carrasco C., Palazuelos J., Guzmán M., Azcoitia I., Galve-Roperh I., Romero J. (2009). Microglial CB2 cannabinoid receptors are neuroprotective in Huntington’s disease excitotoxicity. Brain.

[145] Dowie M.J., Hoffman T., Glass M., Faull R.L., Grimsey N.L. (2014). Cannabinoid receptor CB2 is expressed on vascular cells, but not astroglial cells in the post-mortem human Huntington’s disease brain. J. Chem. Neuroanat..

[146] Denovan-Wright E.M., Kelly M.E., Laprairie R.B. (2013). Cannabinoids increase type 1 cannabinoid receptor expression in a cell culture model of striatal neurons: Implications for Huntington’s disease. Neuropharmacology.

[147] González S., Aroyo I., Benito C., Mechoulam R., Brouillet E., Pazos M.R., Sagredo O., Tolón R.M., Lastres-Becker I., Fernández-Ruiz J. (2008). Cannabinoid CB_2_ receptor agonists protect the striatum against malonate toxicity: Relevance for Huntington’s disease. Glia.

[148] Molinari E., Dowie M.J., Glass M., Scotter E.L. (2010). The therapeutic potential of G-protein coupled receptors in Huntington’s disease. Pharmacol. Ther..

[149] Sinal C.J., Kelly M.E., Rourke J.L., Cairns E.A., Kulkarni P.M., Denovan-Wright E.M., Zrein A., Thakur G.A., Bagher A.M., Laprairie R.B. (2019). Positive allosteric modulation of the type 1 cannabinoid receptor reduces the signs and symptoms of Huntington’s disease in the R6/2 mouse model. Neuropharmacology.

[150] Fernández-Ruiz J., Franco R., Morales P., Jagerovic N., Navarro G., Rodríguez-Cueto C. (2016). Targeting Cannabinoid CB2 Receptors in the Central Nervous System. Medicinal Chemistry Approaches with Focus on Neurodegenerative Disorders. Front. Neurosci..

[151] Musty R., Rein J., Tillery W., Pertwee R., Consroe P. (1997). The Perceived Effects of Smoked Cannabis on Patients with Multiple Sclerosis. Eur. Neurol..

[152] Guaza C., Borrell J., Molina-Holgado E., Vela J.M., Arévalo-Martín Á. (2003). Therapeutic Action of Cannabinoids in a Murine Model of Multiple Sclerosis. J. Neurosci..

[153] Lee T., Mawrin C., Ullrich O., Nitsch R., Raine C.S., Witting A., Eljaschewitsch E., Hoertnagl H., Schneider-Stock R., Schmidt P.M. (2006). The Endocannabinoid Anandamide Protects Neurons during CNS Inflammation by Induction of MKP-1 in Microglial Cells. Neuron.

[154] Correa F., Mestre L., Valenti M., Ortar G., Guaza C., Di Marzo V., Arévalo-Martín A., Molina-Holgado E. (2005). Pharmacological modulation of the endocannabinoid system in a viral model of multiple sclerosis. J. Neurochem..

[155] Correa F., Guaza C., Di Marzo V., Ortega-Gutiérrez S., Molina-Holgado E., Arévalo-Martín Á., Viso A., López-Rodríguez M.L. (2005). Activation of the endocannabinoid system as a therapeutic approach in a murine model of multiple sclerosis. FASEB J..

[156] Mestre L., Docagne F., Correa F., Loría F., Hernangómez M., Borrell J., Guaza C. (2009). A cannabinoid agonist interferes with the progression of a chronic model of multiple sclerosis by downregulating adhesion molecules. Mol. Cell. Neurosci..

[157] Correa F., Guaza C., Mestre L., Docagne F., Clemente D. (2006). The synthetic cannabinoid WIN 55,212-2 increases COX-2 expression and PGE2 release in murine brain-derived endothelial cells following Theiler’s virus infection. Biochem. Pharmacol..

[158] Mecha M., Borrell J., Guaza C., Iñigo P.M., Correa F.G., Hernangómez-Herrero M., Mestre L., Docagne F., Loría F. (2011). Anandamide inhibits Theiler’s virus induced VCAM-1 in brain endothelial cells and reduces leukocyte transmigration in a model of blood brain barrier by activation of CB1receptors. J. Neuroinflammation.

[159] Guaza C., Mecha M., Iñigo P., Mestre L., Feliú A., Carrillo-Salinas F. (2013). Cannabidiol provides long-lasting protection against the deleterious effects of inflammation in a viral model of multiple sclerosis: A role for A2A receptors. Neurobiol. Dis..

[160] Guaza C., Molina-Holgado E., Arevalo-Martin A. (2012). A CB1/CB2 receptor agonist, WIN 55,212-2, exerts its therapeutic effect in a viral autoimmune model of multiple sclerosis by restoring self-tolerance to myelin. Neuropharmacology.

[161] Juknat A., Rimmerman N., Kaushansky N., Vogel Z., Kozela E., Ben-Nun A. (2013). Cannabinoids Decrease the Th17 Inflammatory Autoimmune Phenotype. J. Neuroimmune Pharmacol..

[162] Murphy Á.C., Lynch M.A., Mills K.H., Lalor S.J. (2010). Infiltration of Th1 and Th17 cells and activation of microglia in the CNS during the course of experimental autoimmune encephalomyelitis. Brain Behav. Immun..

[163] Tan J., Shytle R.D., Ehrhart J., Obregon D., Mori T., Hou H., Sun N., Bai Y., Klein T., Fernandez F. (2005). Stimulation of cannabinoid receptor 2 (CB2) suppresses microglial activation. J. Neuroinflammation.

[164] Morales-Montor J., Hernández-Cervantes R., Méndez-Díaz M., Prospéro-García Ó. (2017). Immunoregulatory Role of Cannabinoids during Infectious Disease. Neuroimmunomodulation.

[165] Hansson O. (2021). Biomarkers for neurodegenerative diseases. Nat. Med..

[166] Maccarrone M. (2006). Fatty Acid Amide Hydrolase: A Potential Target for Next Generation Therapeutics. Curr. Pharm. Des..

[167] Kumar A., Shyam H., Kushwaha J., Kumar S., Mishra A., Jain M., Singh M.K. (2021). Role of JAK/STAT in the Neuroinflammation and its Association with Neurological Disorders. Ann. Neurosci..

[168] Bari M., Maccarrone M., Fezza F., Battista N., Gasperi V. (2006). New Insights into Endocannabinoid Degradation and its Therapeutic Potential. Mini-Rev. Med. Chem..

[169] Brown Q.B., Kosten T.A., Makriyannis A., Karanian D.A., Bahr B.A. (2005). Dual Modulation of Endocannabinoid Transport and Fatty Acid Amide Hydrolase Protects against Excitotoxicity. J. Neurosci..

[170] Celorrio M., Fernández-Suárez D., Rojo-Bustamante E., Echeverry-Alzate V., Ramírez M.J., Hillard C.J., López-Moreno J.A., Maldonado R., Oyarzábal J., Franco R. (2016). Fatty acid amide hydrolase inhibition for the symptomatic relief of Parkinson’s disease. Brain Behav. Immun..

[171] Bari M., Battista N., Valenza M., Mastrangelo N., Malaponti M., Catanzaro G., Centonze D., Finazzi-Agrò A., Cattaneo E., Maccarrone M. (2013). In vitro and in vivo models of Huntington’s disease show alterations in the endocannabinoid system. FEBS J..

[172] Battista N., Bari M., Tarditi A., Mariotti C., Bachoud-Lévi A.-C., Zuccato C., Finazzi-Agrò A., Genitrini S., Peschanski M., Di Donato S. (2007). Severe deficiency of the fatty acid amide hydrolase (FAAH) activity segregates with the Huntington’s disease mutation in peripheral lymphocytes. Neurobiol. Dis..

[173] Pasquarelli N., Engelskirchen M., Hanselmann J., Endres S., Porazik C., Bayer H., Buck E., Karsak M., Weydt P., Ferger B. (2017). Evaluation of monoacylglycerol lipase as a therapeutic target in a transgenic mouse model of ALS. Neuropharmacology.

[174] Stella N., Cudaback E., Straiker A., Rickman B., Brosnan C., Witting A., Möller T., Chen L., Walter L. (2006). Experimental autoimmune encephalomyelitis disrupts endocannabinoid-mediated neuroprotection. Proc. Natl. Acad. Sci. USA.

[175] Velayudhan L., McGoohan K.L., Bhattacharyya S. (2021). Evaluation of THC-Related Neuropsychiatric Symptoms Among Adults Aged 50 Years and Older. A Systematic Review and Metaregression Analysis. AMA Netw Open.

